# Effects of traditional Chinese mind-body exercise-Baduanjin for type 2 diabetes on psychological well-being: A systematic review and meta-analysis

**DOI:** 10.3389/fpubh.2022.923411

**Published:** 2022-07-28

**Authors:** Lingjun Kong, Jun Ren, Sitong Fang, Tianxiang He, Xin Zhou, Min Fang

**Affiliations:** ^1^Yueyang Hospital of Integrated Traditional Chinese and Western Medicine, Shanghai University of Traditional Chinese Medicine, Shanghai, China; ^2^Institute of Tuina, Shanghai Institute of Traditional Chinese Medicine, Shanghai, China; ^3^Shuguang Hospital, Shanghai University of Traditional Chinese Medicine, Shanghai, China

**Keywords:** mind-body exercises, Baduanjin, type 2 diabetes, psychological well-being, mental health

## Abstract

**Background:**

Type 2 diabetes is considered one of the most psychologically demanding chronic conditions. Patients suffering from this disease often have poor psychological well-being due to emotional stress. Baduanjin exercises, a traditional Chinese mind-body exercise, are used in the management of type 2 diabetes, especially for mental health. However, the effect of Baduanjin exercises on psychological well-being of patients with type 2 diabetes maintains controversial. Therefore, this systematic review was conducted to evaluate the effects on psychological well-being of Baduanjin exercises for type 2 diabetes.

**Methods:**

Six electronic databases were searched from their inception to March 2022 for randomized controlled trials of Baduanjin exercises for type 2 diabetes. Two reviewers independently extracted data and assessed methodological quality based on PEDro scale. The subgroup analysis was conducted based on different control interventions. The Cochran Q statistic and *I*^2^ were applied to assess the heterogeneity.

**Results:**

Twenty-seven studies between 2005 and 2019 were included in our review. Most of them exceeded the cutoff score 6 based on the PEDro scale. In psychological well-being, the aggregated results indicated that Baduanjin exercises showed positive effects in psychological well-being (*SMD*, 0.96; 95% *CI*, 0.57 to 1.36; *p* < 0.00001), depression (*SMD*, 1.03; 95% *CI*, 0.08 to 1.97; *p* = 0.03), anxiety (*SMD*, 0.88; 95% *CI*, 0.30 to 1.46; *p* = 0.003), and mental health (*SMD*, 0.72; 95% *CI*, 0.42 to 1.02; *p* < 0.00001). In glycemic control, Baduanjin exercises showed better improvements in FBG (*SMD*, 0.53; 95% *CI*, 0.34 to 0.72; *p* < 0.00001), HbA1c (SMD, 0.58; 95% *CI*, 0.41 to 0.75; *p* < 0.00001), and 2-hPBG (*SMD*, 0.56; 95% *CI*, 0.08 to 1.03; *p* = 0.02) compared with usual care/education. However, Baduanjin exercises only showed better improvements in HbA1c when compared with other exercises.

**Conclusions:**

The traditional Chinese mind-body exercise-Baduanjin is a beneficial comprehensive therapy for type 2 diabetes, especially in promoting psychological well-being.

**Systematic review registration:**

https://www.crd.york.ac.uk/PROSPERO/display_record.php?RecordID=110034.

## Introduction

The increasing prevalence of type 2 diabetes has become a global public health concern that results in a substantial socioeconomic and healthcare burden (1). In China, the prevalence of diabetes is as high as 10.9% with many people remaining undiagnosed (2). Diabetes can cause health complications such as cardiovascular disease, nephropathy, and retinopathy. Furthermore, diabetes and its complications have a huge impact on the patients' psychological well-being (3). Type 2 diabetes is one of the most psychologically demanding chronic conditions, and patients often have poor psychological well-being due to emotional stress (4). Depression was associated with poorer glycemic control and the management of its complications in patients with type 2 diabetes (5).

Exercise has generally been recommended in the management of diabetes to reduce the risk factors associated with diabetes such as obesity, sedentary lifestyle, and a high fat diet (6). Low-to-moderate daily exercises are recognized to contribute to glycemic control, psychological well-being, and the management of the complications of type 2 diabetes (7). In China, Baduanjin exercises usually are used as the complementary therapy by the patients with type 2 diabetes, which include eight movements: “holding the hands high with palms up to regulate the internal organs,” “posing as an archer shooting both left- and right-handed,” “holding one arm aloft to regulate the functions of the spleen and stomach,” “looking backwards to prevent sickness and strain,” “swinging the head lowering the body to relieve stress,” “moving the hands down the back and legs and touching the feet to strengthen the kidneys,” “thrusting the fists and making the eyes glare to enhance strength,” and “raising and lowering the heels to cure diseases.” Compared with conventional exercises, Baduanjin exercises, as the traditional Chinese mind-body exercises, emphasize breathing regulation, musculoskeletal relaxation, and relaxing mindfulness for maximizing both physical and psychological well-being of patients with chronic illnesses.

The previous review indicated that Baduanjin exercises plus conventional drug treatments have positive effects in glycemic control of the patients with type 2 diabetes (8). However, there was no systematic review focusing on psychological well-being of Baduanjin exercises for type 2 diabetes. Good psychological well-being could contribute to glycemic control. Several studies have shown beneficial effects in improving depression and anxiety of patients with type 2 diabetes (9–12), but the evidence on the effects of Baduanjin exercises in improving psychological well-being of patients with type 2 diabetes is still insufficient.

Therefore, the current systematic review evaluated the effects of traditional Chinese mind-body exercise-Baduanjin for patients with type 2 diabetes. It provided evidence-based information for the clinical application of Baduanjin exercises for type 2 diabetes, especially for improving psychological well-being.

## Methods

### Trial registration

This systematic review was prospectively registered in PROSPERO with the number CRD42018110034.

### Search strategy

The following electronic databases were searched from their inceptions to March 2022: PubMed, EMBASE, Cochrane Library, China Knowledge Resource Integrated Database (CNKI), Weipu Database for Chinese Technical Periodicals, and Wanfang Data Information. The search terms were diabetes, Baduanjin, eight-brocade exercises, eight-treasured exercises, and eight section brocades. The literature was obtained from other sources: the reference lists of the related reviews, unpublished dissertations, and manual searching at the library of Shanghai University of Traditional Chinese Medicine. There were no restrictions on language or publication status.

### Study selection

In this review, the eligibility criteria for included studies were: (a) randomized clinical trials (RCTs). (b) participants diagnosed with type 2 diabetes, there were no limitations on age, gender, or nationality. (c) Baduanjin exercises were used as a complementary therapy for type 2 diabetes, and all participants received the conventional drugs of type 2 diabetes. The control interventions included usual care, education, and any complementary and alternative therapies without Baduanjin exercises. (d) the primary outcomes included psychological well-being assessed by the Global well-being schedule (GWB) and WHO-5 well-being index (WHO-5); depression assessed by Hamilton depression scale (HAMD), Montgomery Asberg depression rating scale (MADRS), and Self-rating depression scale (SDS), and anxiety assessed by Hamilton anxiety scale (HAMA), and Self-rating anxiety scale (SAS). The secondary outcomes were fasting blood glucose (FBG), glycosylated hemoglobin (HbA1c), 2-h postprandial blood glucose (2-hPBG), and quality of life assessed by 36-Item short-form health survey (SF-36), Diabetes specific quality of life (DSQL), and European quality of life 5-dimensions (EQ-5D). The selection of studies was independently conducted by two reviewers (JR and SF) with good agreement (*k* = 0.86, 95% *CI* 0.54 to 1.00). Any disagreements were discussed by all reviewers.

### Data extraction

According to the pre-defined criteria, data extraction was independently performed by two reviewers (LK and XZ) with good agreement (*k* = 0.847, 95% *CI* 0.66 to 1.00). The following information was extracted: first author, year, and country; sample size; duration of disease; mean age; main outcomes; interventions of Baduanjin exercises group; and interventions of the control group. Only the first phase data were extracted in the crossover studies. Any discrepancies were discussed by all reviewers.

### Quality assessment

Two reviewers (LK and XZ) independently assessed the methodological quality of the included studies using the Physiotherapy Evidence Database (PEDro) scale, which was reported to have a fair to good reliability for RCTs of the physiotherapy in systematic reviews. The PEDro scale includes (a) study eligibility criteria specified, (b) random allocation of subjects, (c) concealed allocation, (d) measure of similarity between groups at baseline, (e) subject blinding, (f) therapist blinding, (g) assessor blinding, (h) <15% dropouts, (i) intention-to-treat analysis, (j) between-group statistical comparisons, and (k) point measures and variability data. The PEDro score was calculated by criteria (b) to (k) according to meeting the criteria or not. The previous studies reported that a cut point of 6 using the PEDro scale indicated high-quality studies (13). Any disagreement was resolved by discussion between the reviewers.

### Data synthesis and analysis

The meta-analysis was performed using the Review Manager Version 5.3 software. For continuous data, the changes from baseline were used in the meta-analysis. The standardized mean difference (*SMD*) and 95% confidence intervals (*CIs*) were calculated. A random effects model was used for the better clinical heterogeneity. The Cochran Q statistic (considered to be statistically significant when *p* < 0.10) and *I*^2^ (where *I*^2^ > 30% indicates moderate heterogeneity; *I*^2^ > 50% substantial heterogeneity; and > 75% considerable heterogeneity) were used to assess the heterogeneity. A sensitivity analysis was conducted to explore possible source of heterogeneity when > 75% considerable heterogeneity. The subgroup analysis was conducted based on different control interventions. The risk of publication bias was assessed if more than ten trials were included in the meta-analysis. The *p* < 0.05 was considered to be statistically significant.

## Results

### Search and selection

**Figure 1:**
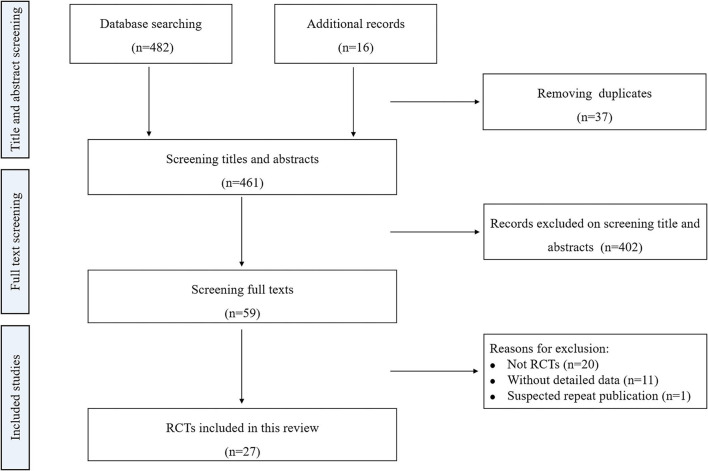
Flow chart for the meta-analysis. RCTs: randomized controlled trials.

The literature search identified 498 records. After removing duplications, 461 potentially relevant studies were identified. A total of 402 studies were excluded after screening the titles and abstracts. We examined 59 full texts for inclusion in our review. After screening the full texts, 32 studies were excluded. Finally, 27 studies were included in our review (10–12, 14–37). The detailed process is summarized in Figure 1.

### Characteristics of the included studies

Twenty-seven studies assessed the effects of Baduanjin exercises, as a complementary therapy, for type 2 diabetes. All studies were conducted in China between 2005 and 2019. A total of 2,048 participants with the mean age of 56.86 ± 6.29 were included in the eligible studies. The diseases duration was 7.53 ± 3.44 years, and control interventions included usual care, education, walking, Tai Chi, and aerobic exercises. The duration of the Baduanjin exercises ranged from 4 to 48 weeks, and almost half of the participants experienced 24 weeks of Banduanjin exercises. The frequency ranged from 2 to 12 sessions per week, and 59% of the studies used 7 sessions per week. The time of each session ranged from 20 to 90 min (43.20 ± 17.77). None assessed the follow-up effect of Baduanjin exercises for type 2 diabetes. The characteristics of the included studies are summarized in Table 1.

**Table 1:** Randomized controlled trials assessing the effect of Baduanjin exercises for type 2 diabetes.

**Study**	**Sample size**	**Mean age (year)**	**Duration of disease (year)**	**Follow-up** **(months)**	**Main outcomes**	**Baduanjin group intervention**	**Control group intervention**
Zhang et al. (14)	25 20	58.4 ± 8.18 58 ± 7.02	10.9 ± 6.84 6.2 ± 6.84	—	FBG, HbA1c, SCL-90, SF-36	Baduanjin (60 min, 3/week, 8 weeks)	Usual care
Wang et al. (15)	40 39	57.8 ± 7.5 56.5 ± 6.9	NR	—	FBG, HbA1c	Baduanjin (60 min, 7/week, 24 weeks)	Usual care
Pan et al. (16)	24 24	47 ± 7 45 ± 9	2.5 ± 1.1 2.6 ± 0.8	—	FBG, HbA1c, BMI, WHR	Baduanjin (45 min, 5/week, 24 weeks)	Usual care
Huang et al. (17)	30 30	57.8 ± 7.5 56.5 ± 6.9	3–10	—	FBG, HbA1c	Baduanjin (60 min, 7/week, 24 weeks)	Usual care
Zhang et al. (10)	15 15	54.9	NR	—	FBG, HbA1c, 2-hPBG, MARDS, HAMA	Baduanjin (60 min, 7/week, 24 weeks)	Usual care
Zhou et al. (18)	63 63	67.4 ± 9.23 68.13 ± 10.64	7.98 ± 1.09 7.87 ± 1.78	—	FBG, HbA1c, 2-hPBG, BMI, WHR	Baduanjin (30 min, 7/week, 12 weeks)	Aerobic exercises (30 min, 3–5/week, 12 weeks)
Duan et al. (19)	100 100	45 Saturday, July 9, 2022 11:12 am± 9 47 ± 7	2.6 ± 0.8 2.5 ± 1.1	—	FBG, HbA1c, BMI, WHR	Baduanjin (20 min, 7/week, 8 weeks)	Aerobic exercises (30 min, 7/week, 8 weeks)
Guan et al. (11)	40 40	59.20 ± 8.80 58.70 ± 8.30	3–18 2–17	—	FBG, HbA1c, 2-hPBG, SAS, SDS	Baduanjin (60 min, 7/week, 24 weeks)	Usual care
Ji et al. (12)	32 30	60.31 ± 7.23 60.26 ± 7.15	NR	—	FBG, HbA1c, 2-hPBG, SAS, SDS, GWB	Baduanjin (45 min, 7/week, 8 weeks)	Education
Liu et al. (20)	33 36	62.64 ± 5.98 65.64 ± 8.38	6.85 ± 5.9 8.25 ± 5.94	—	HbA1c, SDS, SF-36	Baduanjin (40 min, 3–5/week, 12 weeks)	Education (30 min, 1/2 weeks, 12 weeks)
Li et al. (21)	54 54 54 54	54.21 ± 9.47 50.42 ± 9.68 51.62 ± 7.83 52.69 ± 8.37	6.95 ± 3.63 6.02 ± 3.29 6.31 ± 3.53 7.22 ± 4.14	—	FBG, HbA1c, BMI, SF-36	Baduanjin (30 min, 7/week, 36 weeks)	• Aerobic exercise (30 min, 7/week, 36 weeks) • Tai Chi (30 min, 7/week, 36 weeks) • Usual care
Lin et al. (22)	19 19	64.5 ± 11.5 60.8 ± 12.2	2–12 2–9	—	FBG, HbA1c	Baduanjin (90 min, 7/week, 24 weeks)	Usual care
Chen et al. (23)	10 10 10	60.00 ± 5.23 63.70 ± 5.40 63.33 ± 7.78	NR	—	FBG, HbA1c, 2-hPBG, SF-36	Baduanjin (5/ week, 24 weeks)	• Walking (30 min, 14/ week, 24 weeks) • Education
Li et al. (24)	20 20	53.6 ± 8.7 51.4 ± 9.2	7.6 ± 3.6 7.7 ± 3.9	—	FBG, HbA1c, 2-hPBG, PSQI	Baduanjin (30 min, 7/week, 4 weeks)	Usual care
Liu et al. (25)	20 20	57 ± 7 55 ± 9	5.5 ± 1.1 5.6 ± 0.8	—	HbA1c SAS	Baduanjin (30 min, 5/week, 24 weeks)	Usual care
Ren et al. (26)	10 9 9	60.38 ± 4.81 63.12 ± 7.2 60.38 ± 5.81	NR	—	FBG, HbA1c, 2-hPBG, SF-36, BMI, WHR	Baduanjin (5/week, 24 weeks)	• Walking (30 min, 5/week, 24 weeks) • Education
Wei et al. (27)	20 20 20	63.9 ± 7.6 64.8 ± 5.8 65.3 ± 6.0	NR	—	SF-36	Baduanjin (30 min, 5–7/week, 12 weeks)	• Walking (30 min, 5–7/week, 12 weeks) • Usual care
Zhou et al. (28)	13 12	51-72 58-80	0.5–25 1–24	—	FBG, HbA1c, SDS	Baduanjin (30 min, 7/week, 12 weeks)	Usual care
Peng et al. (29)	71 70	NR	NR	—	FBG, SAS, SCL-90	Baduanjin (60 min, 7/week, 24 weeks)	Usual care
Sun et al. (30)	33 32	46.1 ± 11.8	NR	—	FBG, HbA1c, 2-hPBG, SAS, SDS	Baduanjin (60 min, 2/week, 24 weeks)	Aerobic exercises (60 min, 2/week, 24 weeks)
Wang et al. (31)	30 30	61.7 ± 6.9 61.3 ± 8.4	10.6 ± 4.1 10.2 ± 3.6	—	FBG, HbA1c	Baduanjin (20 min, 5/week, 6 weeks)	Usual care
Zhang et al. (32)	45 45 18	57 ± 2.4 55.4 ± 1.70 58.2 ± 1.00	15 ± 0.6 14.5 ± 0.7 14.2 ± 0.5	—	FBG, HbA1c	Baduanjin (30 min, 7/week, 100 days)	• Aerobic exercises (30 min, 7/week, 100 days) • Usual care
Yang et al. (33)	55 55	52.40 ± 2.30 50.30 ± 3.40	11.20 ± 2.10 10.80 ± 1.90	—	HbA1c, HAMD, DSQL	Baduanjin (60 min, 7/week, 12 weeks)	Education (30 min, 6 sessions, 12 weeks)
He et al. (34)	30 28 30	49.63 ± 12.97 52.04 ± 10.60 51.03 ± 11.73	4.29 ± 3.96 4.99 ± 4 4.82 ± 4.05	—	EQ-5D, WHO-5	Baduanjin (20–60 min, 3–7/week, 48 weeks)	• Walking (20-60 min, 3–7/week, 48 weeks) • Education
Liu et al. (35)	45 45	45.01 ± 3.14 43.98 ± 2.81	3.12 ± 1.51 3.26 ± 0.95	—	DSQL	Baduanjin (20 min, 10/week, 12 weeks)	Usual care
Miao et al. (36)	40 40	53.96 ± 5,94 55.56 ± 5.96	NR	—	SF-36	Baduanjin (20 min, 12/week, 12 weeks)	Usual care
Guo et al. (37)	15 15	57.77 ± 6.89 57.53 ± 6.14	10.27 ± 7.63 11.60 ± 6.24	—	FBG, HbA1c, 2-hPBG, BMI, SDSCA	Baduanjin (40–60 min, 4/week, 12 weeks)	Usual care

*BMI, Body mass index; FBG, Fasting blood glucose; DSQL, Diabetes specific quality of life; EQ-5D, European quality of life 5-dimensions; GWB, Globle well-being schedule; HAMA, Hamilton anxiety scale; HAMD, Hamilton depression scale; HbA1c, Gglycosylated haemoglobin; 2-hPBG, 2-h postprandial blood glucose; MADRS, Montgomery asberg depression rating scale; NR, Not reported; PSQI, Pittsburgh sleep quality index; SAS, Self-rating anxiety scale; SDS, Self-rating depression scale; SDSCA, Summary of diabetes self care activities; SCL-90, Symptom checklist 90; SF-36, 36-Item short form health survey; WHO-5, WHO-5 well-being index*.

### Methodological quality

Most studies (89%) of Baduanjin exercises for type 2 diabetes exceeded the PEDro cutoff score of 6. The greatest potential bias was associated with blinding and concealed allocation. Only two studies performed assessor blinding (11, 29) and five studies did not use the intention-to-treat analysis (11, 26, 32, 33, 37). Other items were scored positive in the included studies. The detailed scores were shown in Table 2.

**Table 2:** PEDro scale of quality for the included trials.

**Study**	**Eligibility criteria**	**Random allocation**	**Concealed allocation**	**Similar at baseline**	**Subjects blinded**	**Therapists blinded**	**Assessors blinded**	**<15% dropouts**	**Intention-to-treat analysis**	**Between-group comparisons**	**Point measures and variability data**	**Total**
Zhang et al. (14)	1	1	0	1	0	0	0	1	1	1	1	6
Wang et al. (15)	1	1	0	1	0	0	0	1	1	1	1	6
Pan et al. (16)	1	1	0	1	0	0	0	1	1	1	1	6
Huang et al. (17)	1	1	0	1	0	0	0	1	1	1	1	6
Zhang et al. (10)	1	1	0	1	0	0	0	1	1	1	1	6
Zhou et al. (18)	1	1	0	1	0	0	0	1	1	1	1	6
Duan et al. (19)	1	1	0	1	0	0	0	1	1	1	1	6
Guan et al. (11)	1	1	0	1	0	0	1	1	0	1	1	6
Ji et al. (12)	1	1	0	1	0	0	0	1	1	1	1	6
Liu et al. (20)	1	1	0	1	0	0	0	1	1	1	1	6
Li et al. (21)	1	1	0	1	0	0	0	1	1	1	1	6
Lin et al. (22)	1	1	0	1	0	0	0	1	1	1	1	6
Chen et al. (23)	1	1	0	1	0	0	0	1	1	1	1	6
Li et al. (24)	1	1	0	1	0	0	0	1	1	1	1	6
Liu et al. (25)	1	1	0	1	0	0	0	1	1	1	1	6
Ren et al. (26)	1	1	0	1	0	0	0	1	0	1	1	5
Wei et al. (27)	1	1	0	1	0	0	0	1	1	1	1	6
Zhou et al. (28)	1	1	0	1	0	0	0	1	1	1	1	6
Peng et al. (29)	1	1	0	1	0	0	1	1	1	1	1	7
Sun et al. (30)	1	1	0	1	0	0	0	1	1	1	1	6
Wang et al. (31)	1	1	0	1	0	0	0	1	1	1	1	6
Zhang et al. (32)	1	1	0	1	0	0	0	1	0	1	1	5
Yang et al. (33)	1	1	1	1	0	0	0	1	0	1	1	6
He et al. (34)	1	1	0	1	0	0	0	1	1	1	1	6
Liu et al. (35)	1	1	0	1	0	0	0	1	1	1	1	6
Miao et al. (36)	1	1	0	1	0	0	0	1	1	1	1	6
Guo et al. (37)	1	1	0	1	0	0	0	1	0	1	1	5

*Criteria (2–11) were used to calculate the total PEDro score. Each criterion was scored as either 1 or 0 according to whether the criteria was met or not, respectively*.

### Synthesis of the results

#### Psychological well-being

**Figure 2:**
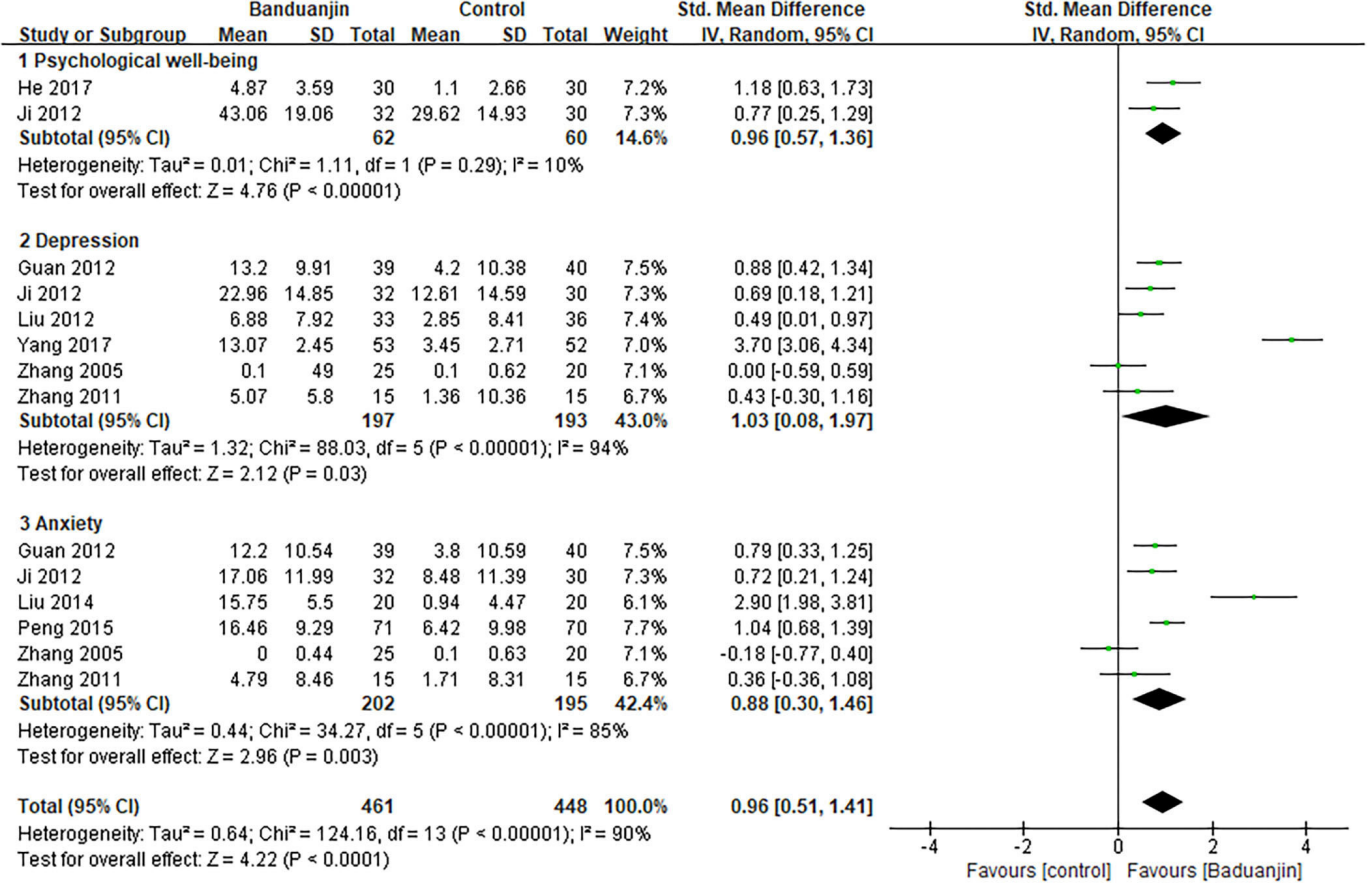
Forest plot of the effects of Baduanjin exercises on psychological well-being.

The aggregated result in the meta-analysis showed that Baduanjin exercises significantly improved the psychological well-being of patients with type 2 diabetes (*SMD*, 0.96; 95% *CI*, 0.51 to 1.41; *p* < 0.0001, Figure 2). In the subgroup analysis, the aggregated results indicated that Baduanjin exercises showed better improvements in psychological well-being (*SMD*, 0.96; 95% *CI*, 0.57 to 1.36; *p* < 0.00001, Figure 2), depression (*SMD*, 1.03; 95% *CI*, 0.08 to 1.97; *p* = 0.03, Figure 2) and anxiety (*SMD*, 0.88; 95% *CI*, 0.30 to 1.46; *p* = 0.003, Figure 2) compared with usual care/education. Sun also reported that 6 months of Baduanjin exercise showed better improvements in depression and anxiety in patients with type 2 diabetes compared with aerobic exercises (30). Sensitivity analyses yielded similar and non-significant differences for the above-mentioned outcomes.

#### Quality of life

**Figure 3:**
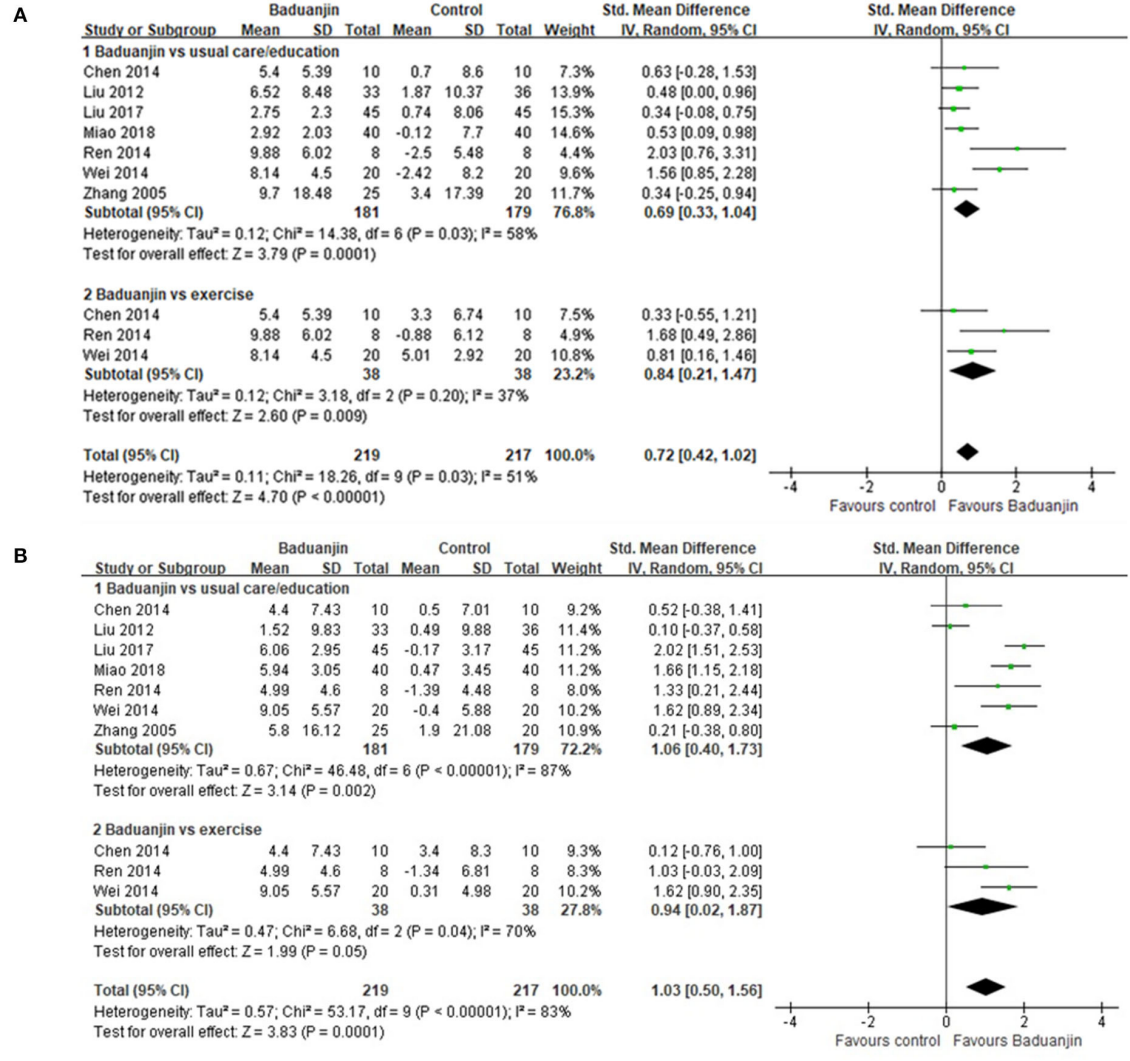
Forest plot of the effects of Baduanjin exercises on quality of life. **(A)** mental component scores, **(B)** physical component scores.

In mental component scores, the aggregated result demonstrated that Baduanjin exercises also significantly improved mental health of the patients with type 2 diabetes (*SMD*, 0.72; 95% *CI*, 0.42 to 1.02; *p* < 0.00001, Figure 3A). In the subgroup analysis, Baduanjin exercises showed better improvements in mental health compared with usual care/education (*SMD*, 0.69; 95% *CI*, 0.33 to 1.04; *p* = 0.0001, Figure 3A) and other exercises (*SMD*, 0.84; 95% *CI*, 0.21 to 1.47; *p* = 0.009, Figure 3A). In physical component scores, Baduanjin exercises showed better improvements in physical health compared with usual care/education (*SMD*, 1.06; 95% *CI*, 0.40 to 1.73; *p* = 0.002, Figure 3B), but not compared with other exercises (*SMD*, 0.94; 95% *CI*, 0.02 to 1.87; *p* = 0.05, Figure 3B). Sensitivity analyses yielded similar and non-significant differences for the above-mentioned outcomes.

#### Glycemic control

**Figure 4:**
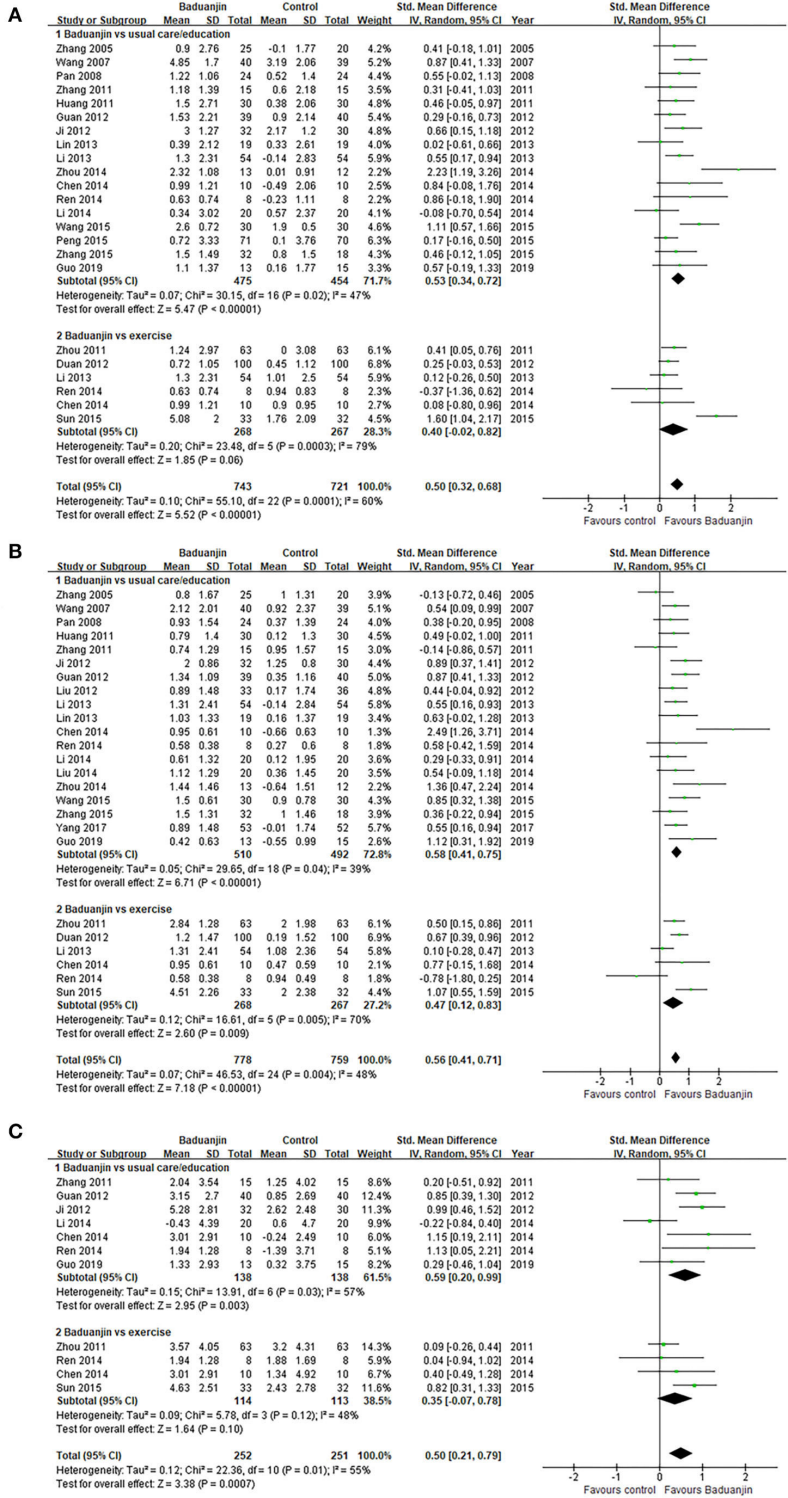
Forest plot of the effects of Baduanjin exercises on Glycemic control. **(A)** fasting blood glucose, **(B)** glycosylated hemoglobin, **(C)** 2-h postprandial blood glucose.

Compared with usual care/education, Baduanjin exercises showed better improvements in FBG (*SMD*, 0.53; 95% *CI*, 0.34 to 0.72; *p* < 0.00001, Figure 4A), HbA1c (*SMD*, 0.58; 95% *CI*, 0.41 to 0.75; *p* < 0.00001, Figure 4B), and 2-hPBG (*SMD*, 0.59; 95% *CI*, 0.20 to 0.99; *p* = 0.003, Figure 4C). However, the aggregated result indicated that the Baduanjin exercises only showed better improvements in HbA1c (*SMD*, 0.47; 95% *CI*, 0.12 to 0.83; *p* = 0.009, Figure 4B) compared with other exercises. Baduanjin exercises did not get better gains in FBG (*SMD*, 0.40; 95% *CI*, −0.02 to 0.82; *p* = 0.06, Figure 4A) or 2-hPBG (*SMD*, 0.35; 95% *CI*, −0.07 to 0.78; *p* = 0.10, Figure 4C) compared with other exercises. Sensitivity analyses yielded similar and non-significant differences for the above-mentioned outcomes.

#### Body fat control

**Figure 5:**
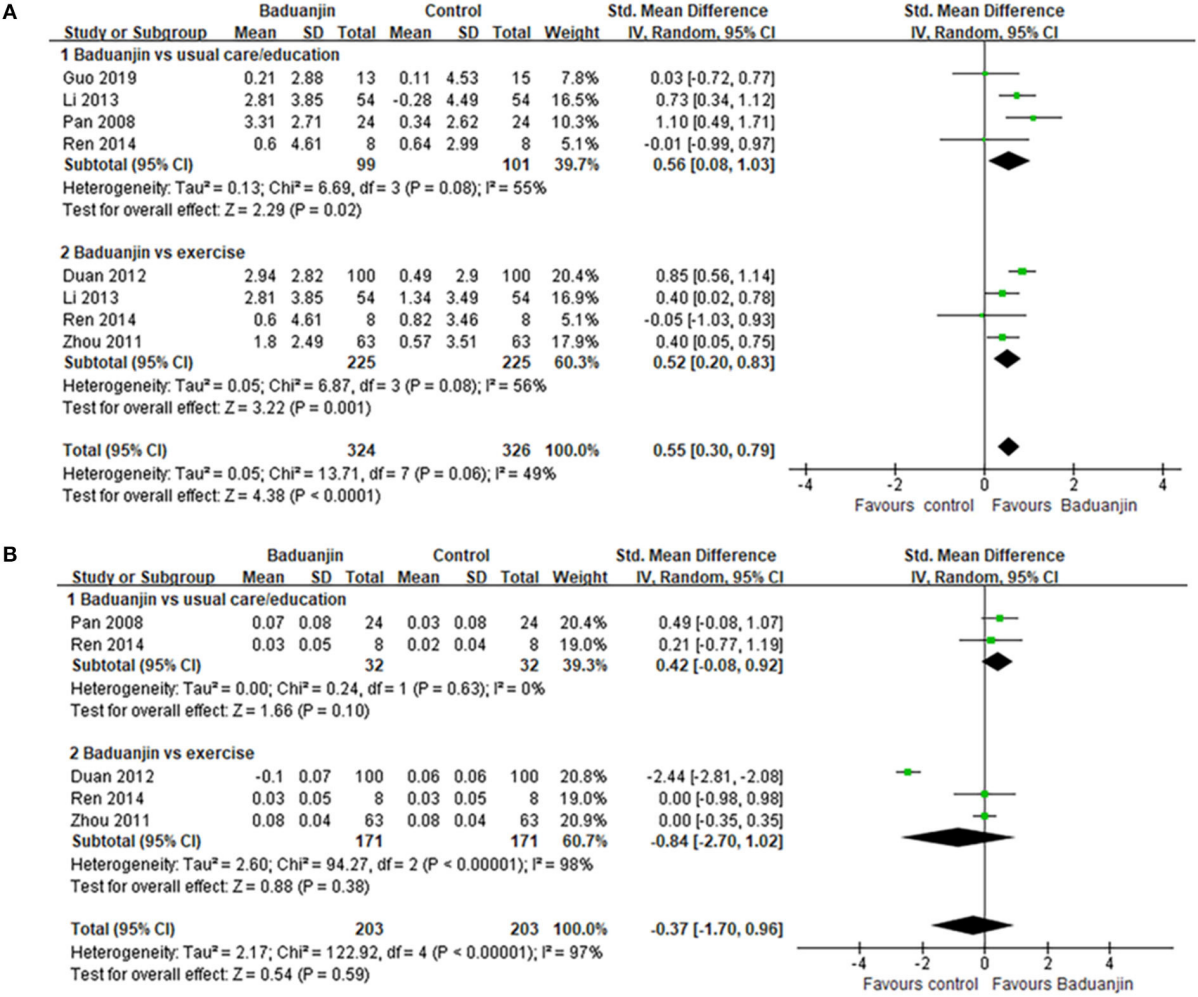
Forest plot of the effects of Baduanjin exercises on **(A)** body mass index and **(B)** wait-hip ratio.

The aggregated result also showed that Baduanjin exercises got better gains on body mass index compared with usual care/education (*SMD*, 0.56; 95% *CI*, 0.08 to 1.03; *p* = 0.02, Figure 5A) and other exercises (*SMD*, 0.52; 95% *CI*, 0.20 to 0.83; *p* = 0.001, Figure 5A). However, Baduanjin exercises did not show better improvements in wait–hip ratio of patients compared with usual care/education (*SMD*, 0.42; 95% *CI*, −0.08 to 0.92; *p* = 0.10, Figure 5B) or other exercises (*SMD*, −0.84; 95% *CI*, −2.70 to 1.02; *p* = 0.38, Figure 5B). Sensitivity analyses yielded similar and non-significant differences for the above-mentioned outcomes.

#### Publication bias

**Figure 6:**
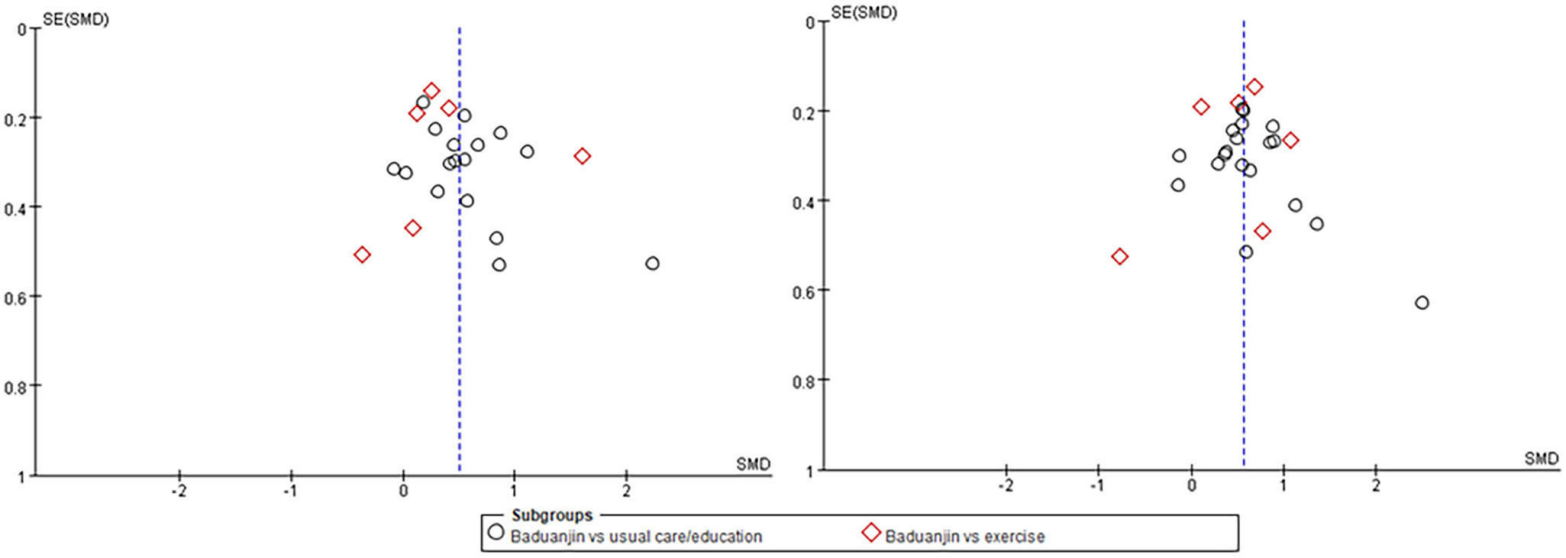
The funnel plots for Badunjin exercises for type 2 diabetes in fasting blood glucose (left) and glycosylated hemoglobin (right).

The funnel plots for Badunjin exercises for type 2 diabetes in FBG and HbA1c including 20 RCTs, respectively, are shown in Figure 6. The publication bias was small because the spots were substantially symmetric.

#### Adverse events

None of the included studies reported whether there were adverse events or not.

## Discussion

To our knowledge, the current study is the first systematic review that attempts to summarize and examines the effects on the psychological well-being of Baduanjin exercises, as the complementary therapy, for type 2 diabetes. The current results demonstrate that Baduanjin exercises can significantly improve the psychological well-being of patients with type 2 diabetes such as depression, anxiety, mental health, and global well-being. In glycemic control, Baduanjin exercises showed better improvements than usual care/education, but not other exercises. However, there was insufficient evidence on the long-term effects of Baduanjin exercises for type 2 diabetes.

The previous review reported that evidence from meta-analysis suggested that Baduanjin exercises plus conventional therapy have positive effects on the type 2 diabetes (8). In the included studies, conventional therapy included training, regular drug treatment, relaxation exercises, and regular healthcare. The control interventions included regular healthcare, regular drug treatment, and aerobics. However, the detailed subgroup analysis was not conducted based on different conventional therapy and control intervention. Furthermore, the effects on the psychological well-being of Baduanjin exercises for type 2 diabetes were not focused on the previous review. Baduanjin exercises, as the traditional Chinese mind-body exercises, have positive effects on anxiety and depression of patients with type 2 diabetes (10–12). Good psychological well-being could contribute to glycemic control. In our review, the study of Baduanjin exercises plus other exercises was not included. The detailed subgroup analysis was conducted based on different control interventions. And the current review showed the evidence of the effects on the psychological well-being of Baduanjin exercises, as the complementary therapy, for type 2 diabetes. Therefore, there was more powerful evidence on glycemic control and promoting psychological well-being of Baduanjin exercises for type 2 diabetes in our review.

Most included studies reported the beneficial effect on the quality of life and psychological well-being of Baduanjin exercises for type 2 diabetes. The results also supported the positive effects of Baduanjin exercises on improving depression, anxiety, mental health, and global well-being of patients with type 2 diabetes. Baduanjin exercises can effectively ameliorate the symptoms of depression and blood glucose levels of patients with type 2 diabetes possibly by regulating the dysregulated expression of lncRNA, mRNA, and circRNA (38). Compared with the conventional exercises focusing on strengthening physical health, Baduanjin exercises, as a traditional Chinese mind-body exercise, emphasize breathing regulation, an empty state of mindfulness, and musculoskeletal relaxation in order to maximize both physical and psychological well-being. These mindfulness-based elements are very important in reducing anxiety and depression (39). The rhythmic breathing regulation has a calming effect on stress-related behaviors (40). The previous study indicated that the effect in reducing depression of Baduanjin exercises was associated with increased levels of plasma adiponectin, which has an antidepressant-like function (41). The previous review also indicated positive effects in the quality of life and mental health of Baduanjin exercises for college students, elderly, and patients with chronic physical illnesses and mental problems (42). Li et al. (24) reported that Baduanjin exercises improved the sleep quality of type 2 diabetes that may promote psychological well-being.

In glycemic control, Baduanjin exercises, as a complementary therapy, showed better effects in FBG, HbA1c, and 2-hPBG than usual care/education. Based on the theories of traditional Chinese medicine, Baduanjin exercises could dredge meridians and collaterals, facilitate blood circulation, and regulate the internal organs to enhance one's health. The review also reported that Baduanjin exercises could modulate the blood lipid profile by decreasing total cholesterol, triglyceride, and low density lipoprotein cholesterol levels, and increasing high density lipoprotein cholesterol levels (43). However, the potential mechanism of Baduanjin exercises in modulating the blood glucose and lipid is unclear.

Compared with other exercises, Baduanjin exercises only showed better improvements in HbA1c, not in FBG or 2-hPBG. It may be associated with the low intensity and speed of Baduanjin exercises. As a mind-body exercise, Baduanjin practitioners pay more attention to unifying movements, breathing, and mindfulness for maximizing mind-body well-being. Therefore, the appropriate exercise intensity may get better health benefits from Baduanjin exercises. However, there were obvious differences in exercise time and frequency of Baduanjin exercises in the included studies. The long-term Baduanjin exercise may get more benefits for type 2 diabetes because 55% included studies had more than 24 weeks exercises. In the further, detailed dose-effect study of Baduanjin exercises for type 2 diabetes is warranted.

### Limitations

In our review, some limitations should be considered. There were no restrictions in publication languages, but all of the included studies were published in Chinese. Therefore, Baduanjin exercises are not popular worldwide, and more studies and reports of Baduanjin exercises for type 2 diabetes are warranted. The methodological limitations of the eligible RCTs may also exaggerate the current results because the majority of the studies did not use concealed allocation and blinding. The blinding of therapists and participants is difficult for the studies of complementary and alternative therapies, such as Baduanjin exercises, but concealed allocation and blinding of assessors should be performed. In addition, there was high heterogeneity in some meta-analyses. Sensitivity analyses yielded similar and non-significant differences for the results. No clear source of heterogeneity was found. It may be due to the different frequency, duration, and session of Baduanjin exercises. In the included studies, the duration of Baduanjin exercises ranged from 4 to 48 weeks, the frequency ranging from 2 to 12 sessions per week, and the time of each session ranging from 20 to 90 min. Finally, no studies reported adverse effects of Baduanjin exercises for type 2 diabetes; but we could not confirm its safety.

## Conclusions

The current review demonstrates that Baduanjin exercises can significantly improve psychological well-being of the patients with type 2 diabetes. There was beneficial evidence on glycemic control of Baduanjin exercises for type 2 diabetes. There was insufficient evidence on the long-term effects of Baduanjin exercises for type 2 diabetes; but traditional Chinese mind-body exercise-Baduanjin should be considered as a beneficial comprehensive therapy for type 2 diabetes, especially in promoting psychological well-being. Possible effective Baduanjin exercises are 30 min/day during 24 consecutive weeks based on the characteristics of the included studies.

There is further need of studies to assess the long-term effects of Baduanjin exercises for type 2 diabetes. Future research should explore the potential mechanisms by which Baduanjin exercises improve psychological well-being and glycemic control for type 2 diabetes.

## Data availability statement

The original contributions presented in the study are included in the article/supplementary material, further inquiries can be directed to the corresponding authors.

## Author contributions

LK, JR, XZ, and MF conserved, designed the study, and wrote the draft manuscript. TH and JR performed the literature search. JR and SF identified and selected the studies. LK and XZ assessed the methodological quality and extracted data. XZ and MF performed data synthesis and analysis. All authors contributed to the article and approved the submitted version.

## Funding

This work was supported by the Shanghai Pujiang Program (21PJD071), Talent development program in Shanghai (2019048), and Innovation Team and Talents Cultivation Program of National Administration of Traditional Chinese Medicine (ZYYCXTD-C-202008).

## Conflict of interest

The authors declare that the research was conducted in the absence of any commercial or financial relationships that could be construed as a potential conflict of interest.

## Publisher's note

All claims expressed in this article are solely those of the authors and do not necessarily represent those of their affiliated organizations, or those of the publisher, the editors and the reviewers. Any product that may be evaluated in this article, or claim that may be made by its manufacturer, is not guaranteed or endorsed by the publisher.
